# Severe diffuse alveolar hemorrhage related to autoimmune disease: a multicenter study

**DOI:** 10.1186/s13054-020-02936-0

**Published:** 2020-05-18

**Authors:** Adrien Mirouse, Antoine Parrot, Vincent Audigier, Alexandre Demoule, Julien Mayaux, Guillaume Géri, Eric Mariotte, Nicolas Bréchot, Nicolas de Prost, Mathieu Vautier, Mathilde Neuville, Naïke Bigé, Etienne de Montmollin, Patrice Cacoub, Matthieu Resche-Rigon, Jacques Cadranel, David Saadoun

**Affiliations:** 1 Département Hospitalo-Universitaire Inflammation-Immunopathologie-Biotherapie (DHU i2B), UPMC Université Paris 06, UMR 7211, Sorbonne Universités, 75005 Paris, France; 2 INSERM, UMR_S 959, 75013 Paris, France; 3 CNRS, FRE3632, 75005 Paris, France; 4 Département de Médecine Interne et Immunologie Clinique, National Center for Autoimmune and Systemic Diseases and National Center for Autoinflammatory Diseases and Amyloidosis, APHP, Groupe Hospitalier Pitié-Salpêtrière, 75013 Paris, France; 5 Service de Pneumologie, Hôpital Tenon, APHP, Paris, France; 6 Département de Biostatistiques, Hôpital Saint-Louis, APHP, Paris, France; 7 Service de Réanimation Médicale et Pneumologie, Hôpital Pitié-Salpêtrière, APHP, Paris, France; 8 Service de Réanimation Médicale, Hôpital Cochin, APHP, Paris, France; 9 Service de Réanimation Médicale, Hôpital Saint-Louis, APHP, Paris, France; 10 Service de Réanimation Médicale, Hôpital Pitié-Salpêtrière, APHP, Paris, France; 11 Service de Réanimation Médicale, Hôpital Henri Mondor, APHP, Créteil, France; 12 Service de Médecine Interne, CHU de Caen, Caen, France; 13 Service de Réanimation Médicale, Hôpital Bichat, APHP, Paris, France; 14 Service de Réanimation Médicale, Hôpital Saint-Antoine, Paris, France; 15 Service de Réanimation Medico-chirurgicale, Hôpital Delafontaine, Saint-Denis, France

**Keywords:** Diffuse alveolar hemorrhage, ICU, mechanical ventilation, Plasma exchange, ANCA-associated vasculitis, Anti-MBG-associated vasculitis, IgA-associated vasculitis, Cryoglobulinemia, Systemic lupus erythematosus, Antiphospholipid syndrome

## Abstract

**Background:**

Diffuse alveolar hemorrhage (DAH) occurs during the course of autoimmune disease and may be life threatening. The objective was to assess characteristics and prognosis factors of DAH who required intensive care unit (ICU) admission in patients with autoimmune diseases.

**Methods:**

French multicenter retrospective study including patients presenting DAH related to autoimmune diseases requiring ICU admission from 2000 to 2016.

**Results:**

One hundred four patients (54% of men) with median age of 56 [32–68] years were included with 79 (76%) systemic vasculitis and 25 (24%) connective tissue disorders. All patients received steroids, and 72 (69%), 12 (11.5%), and 57 (55%) patients had cyclophosphamide, rituximab, and plasma exchanges, respectively. During ICU stay, 52 (50%), 36 (35%), and 55 (53%) patients required mechanical ventilation, vasopressor use, and renal replacement therapy, respectively. Factors associated with mechanical ventilation weaning were age (HR [95%CI] 0.97 [0.96–0.99] per 10 years, *p* < 0.0001), vasculitis-related DAH (0.52 [0.27–0.98], *p* = 0.04), and time from dyspnea onset to ICU admission (0.99 [0.99–1] per day, *p* = 0.03). ICU mortality was 15%. Factors associated with alive status at ICU discharge were chronic cardiac failure (HR [95%CI] 0.37 [0.15–0.94], *p* = 0.04), antiphospholipid syndrome-related DAH (3.17 [1.89–5.32], *p* < 0.0001), SAPS II (0.98 [0.97–0.99], *p* = 0.007), and oxygen flow at ICU admission (0.95 [0.91–0.99] per liter/min, *p* = 0.04).

**Conclusion:**

DAH in autoimmune diseases is a life-threatening complication which requires mechanical ventilation in half of the cases admitted to ICU.

## Background

Diffuse alveolar hemorrhage (DAH) is a form of pulmonary hemorrhage that originates from the pulmonary microcirculation [1]. DAH is usually classified as immune or non-immune [2]. Immune causes represent 30 to 40% of all DAH cases [3]. Autoimmune diseases associated with DAH include mainly small-vessel vasculitis, systemic lupus erythematosus (SLE), and antiphospholipid syndrome (APS). Immune DAH severity is variable. It can range from asymptomatic forms, diagnosed on systematic chest radiography, to life-threatening complications. Severe forms may lead to acute respiratory failure and require intensive care unit (ICU) management [4]. DAH represents 12% of ICU admissions in patients with an autoimmune disease [5].

Early recognition and diagnosis are mandatory to initiate adequate treatments. Treatment is based on high-dose steroids in combination with cyclophosphamide or rituximab [6–8]. However, in this indication, these treatment regimens are based on case reports and small cohorts with immune DAH of heterogeneous severity. Plasma exchange therapy (PLEX) has been reported as efficient in several case series [9, 10]. PLEX has been reported as efficient in ANCA-associated vasculitis concerning 1-year renal outcome [11, 12]. These results were not confirmed in a recent phase III study in which plasma exchanges were not associated with end-stage renal disease rate reduction in ANCA-associated vasculitis [13].

Data regarding prognosis and outcome of severe DAH in autoimmune diseases are lacking as these patients are often excluded from large clinical trials [14–16]. In this French multicenter study, we aimed to assess the outcome and prognosis factors of severe DAH in autoimmune diseases and to compare vasculitis- and connective tissue disorder-related DAH.

## Methods

### Design

We conducted a retrospective French multicenter study in 11 ICU. This study was approved by the local ethics committee. All adult patients (≥ 18 years old) with a severe DAH related to an autoimmune disease admitted in the participating ICUs between January 1, 2000, and January 1, 2017, were included. Patients were identified with the International Disease Classification 10 code for auto-immune diseases (lupus, vasculitis, inflammatory myopathy, systemic sclerosis, overlap autoimmune diseases, rheumatoid arthritis) and DAH. DAH was defined as (1) hemoptysis and/or macroscopically hemorrhagic broncho-alveolar lavage, (2) new pulmonary infiltrates, and (3) anemia. DAH was defined as severe if DAH management required ICU admission. Autoimmune diseases included were small-vessel vasculitis and connective tissue disorders. Autoimmune diseases were diagnosed according to international diagnosis criteria [17–19]. Patients were excluded if DAH at admission was related to a non-immune condition such as heart failure or infection. Patients were identified from the ICU databases using codes for acute respiratory failure and for autoimmune diseases. All patients’ medical records were reviewed by 2 investigators (AM and DS).

### Data collection

Demographic data, comorbidities, treatments, medical history, and clinical, biological, and radiological findings were abstracted from medical charts. Comorbidities were assessed with the Charlson comorbidity index [20]. Autoimmune diseases manifestations at ICU admission were collected including skin, neurological, joint, gut, and kidney manifestations. Physiological variables, laboratory data, and radiographic findings (chest X-ray and computed tomography [CT] when available) at ICU admission were also reported. Disease severity was assessed using the Simplified Acute Physiology Score (SAPS II) [21]. Patients were classified as having acute respiratory failure if they met the following criteria: severe dyspnea at rest, respiratory rate greater than 30 breaths per minute or clinical signs of respiratory distress, and oxygen saturation less than 92% or PaO2 less than 60 mmHg on room air [22]. Hypoxemia severity was assessed using the PaO_2_/FiO_2_ ratio. Fiberoptic bronchoscopy, bronchoalveolar lavage, and the use of life-sustaining treatments (i.e., noninvasive or invasive mechanical ventilation, renal replacement therapy, and vasopressors) were recorded. Diagnosis of the autoimmune disease was made according to the clinical, biological, immunological, and histological findings. Acute respiratory distress syndrome (ARDS) was defined according to the Berlin definition [23]. Acute kidney injury was defined according to the Kidney Disease Improving Global Outcomes definitions [24]. Therapeutic regimens were reported including red blood cell transfusions, standard dose steroids, high-dose steroid pulses, other immunosuppressive therapies, and plasma exchange therapy. ICU-acquired infections were recorded. The diagnosis of infection was confirmed if patients met both following criteria: microbiological identification of a pathogen and administration of related antibiotic treatment.

Mechanical ventilation weaning was defined as extubation without reintubation during the following 48 h. ICU and hospital length of stays and vital status at ICU and hospital discharge were obtained for all patients.

### Statistics

Patients’ characteristics were described using medians and interquartile ranges for quantitative variables and counts and percentages for qualitative variables. Characteristics of patients requiring mechanical ventilation during their ICU stay were compared to those of patients without mechanical ventilation using either the Wilcoxon rank sum test or Fishers’ exact test. Comparisons between patients having received plasma exchanges and not, between those being deceased and being discharged alive, and between those having developed a vasculitis and a connective tissue disorder were performed in the same way. To assess variables associated with the weaning of mechanical ventilation, clinical relevant baseline characteristics significantly associated with the weaning from mechanical ventilation, as well as treatments provided during ICU stay, were included in a multivariate Cox model, where treatment variables are time-varying covariates and death in ICU is considered as a competing event of discharge alive from ICU. The same procedure was used for assessing variables associated with ICU discharge.

## Results

### Clinical characteristics of DAH

During the study period, we identified 104 patients (54% of men) admitted to the ICU for severe DAH associated with autoimmune disease (Table 1 and supplementary e-Table 1). The median age was 56 [32–68] years old. DAH was associated with an autoimmune disease relapse in 28 (27%) patients. DAH was the first autoimmune disease manifestation in 35 (34%) patients. A pneumo-renal syndrome was present in 83 (81%) patients. The median time from onset of respiratory symptoms to ICU admission was 5 [1–21] days. On admission, patients were severely hypoxemic with a PaO_2_/FiO_2_ ratio of 150 [87–229] mmHg. Hemoptysis was present in 53 (51%) patients, and acute respiratory failure was reported in 78 (75%) patients. Lactate dehydrogenase (LDH) elevation was present in 65 (63%) patients. Bronchoscopy was performed in 83 (80%) patients and demonstrated macroscopic pulmonary hemorrhage in all cases.

**Table 1:** Characteristics of 104 patients with DAH and according to the presence of a vasculitis or a connective tissue disorder

	All patients (***n*** = 104)	Connective tissue disorder (***n*** = 25)	Vasculitis (***n*** = 79)	***p***
**A. Baseline characteristics**				
**Demographics**				
Age, years, median [IQR]	56 [32–68]	42 [27–55]	61 [39–71]	0.003
Male gender, *n* (%)	56 (54%)	7 (29%)	48 (61%)	0.02
**Comorbidities**				
Charlson score, median [IQR]	3 [1–4]	2 [1–3.5]	3 [1–4]	0.23
Previous steroid treatment, *n* (%)	26 (25%)	10 (40%)	16 (20%)	0.064
**Systemic disease,*n*(%)**				
Connective tissue disorder	25 (24%)	NA	NA	
Systemic lupus erythematosus	12 (48%)			
Primary antiphospholipid syndrome	9 (36%)			
Others*	4 (16%)			
Vasculitis	79 (76%)	NA	NA	
ANCA-associated vasculitis	57 (72%)			
Anti-GBM disease	12 (15%)			
Cryoglobulin-associated vasculitis	4 (5%)			
IgA-associated vasculitis	6 (8%)			
Inaugural	76 (73%)	13 (52%)	63 (80%)	0.01
Relapse	28 (27%)	12 (48%)	16 (20%)	
**Systemic disease manifestations, n (%)**				
DAH	104 (100%)	25 (100%)	79 (100%)	1
Hemoptysis	53 (51%)	10 (40%)	43 (54%)	0.25
Cough	68 (65%)	17 (68%)	51 (65%)	0.81
Acute respiratory failure	78 (75%)	20 (80%)	58 (73%)	0.6
Renal	83 (81%)	14 (56%)	69 (89%)	0.0009
Digestive	7 (7%)	2 (8%)	5 (6%)	0.68
Nervous system	16 (16%)	6 (24%)	10 (13%)	0.21
Joint	20 (19%)	4 (16%)	16 (21%)	0.78
Skin	23 (22%)	7 (28%)	16 (21%)	0.42
**ICU admission characteristics**, median [IQR]				
SAPS II	36 [25.75–47]	33 [26–42]	38 [26–48]	0.51
Temperature	37.9 [37–38.6]	38.2 [37.2–39.1]	37.7 [37–38.5]	0.15
Oxygen flow, L/min	15 [5–15]	12 [5–15]	15 [5–15]	0.92
PaO2/FiO2 ratio	150 [87–229]	120 [90–230]	157 [86–225]	0.64
Time (days) from dyspnea onset to ICU admission	5 [1–21]	1 [0–10]	6 [1–24]	0.018
Time (days) from first symptoms to ICU admission	30 [11.8–79]	17 [10–46]	32 [15–88]	0.079
Time (days) from hospital admission to ICU admission	5 [1–12]	7 [3–15]	3 [0–12]	0.13
**Biological findings at ICU admission**, median [IQR]				
Hemoglobin, g/L	86 [72–95]	87 [75–97]	86 [72–95]	0.73
Leukocytes, G/L	11.7 [8.9–15.6]	11.1 [6.4–17.1]	11.7 [9.2–15.4]	0.47
Platelets count, G/L	257 [141–353]	102 [53–236]	278 [186–401]	< 0.0001
Creatinine, μmol/L	235 [92–433]	103 [71–174]	303 [128–520]	0.0002
Urine protein/creatinine, g/mmol	0.19 [0.1–0.33]	0.12 [0.07–0.25]	0.2 [0.1–0.34]	0.17
LDH, mmol/L	488 [354–905]	873 [532–1152]	454 [329–714]	0.008
**B. ICU management.**				
**ICU management,*n*(%)**				
Vasopressor use	36 (35%)	8 (32%)	28 (35%)	0.81
Renal replacement therapy	55 (53%)	6 (24%)	49 (62%)	0.001
Respiratory management				
Non-invasive ventilation	33 (32%)	8 (32%)	25 (32%)	1
Mechanical ventilation	52 (50%)	11 (44%)	41 (52%)	0.65
ARDS diagnosis	52 (50%)	13 (52%)	43 (55%)	0.82
Prone positioning	11 (21%)	3 (12%)	8 (11%)	1
Mechanical ventilation duration, days, median [IQR]	12 [6–22]	4 [4–18]	13 [9–33]	0.007
Red blood cell transfusion	78 (75%)	16 (64%)	62 (80%)	0.33
**Systemic disease management,*n*(%)**				
Time (days) from first symptoms to diagnosis	31 [11.5–77]	19.5 [10–53]	32 [15–86]	0.14
Steroids	103 (99%)	25 (100%)	78 (99%)	1
Steroids pulse therapy	93 (89%)	20 (80%)	73 (92%)	0.13
Cyclophosphamide	72 (69%)	9 (36%)	63 (80%)	< 0.0001
Rituximab	12 (11.5%)	1 (4%)	11 (14%)	0.29
PLEX	57 (55%)	6 (24%)	51 (65%)	0.0009
Number of PLEX	7 [4.3–9.5]	4 [2–9]	7 [5–9]	0.32
**Bacterial superinfection,*n*(%)**	30 (29%)	3 (12%)	27 (34%)	0.042
**Hospital mortality,*n*(%)**	16 (15%)	3 (12%)	13 (17%)	0.76
**Follow-up**	48 (55%)	13 (59%)	35 (53%)	0.65
Length of follow-up (months)	19 [8–38]	37 [11–66]	15 [7–28]	0.003
Chronic renal failure**	22 (65%)	4 (80%)	18 (62%)	0.33
Dialysis**	8 (24%)	1 (20%)	7 (24%)	0.42

*Other connective tissue disorders: mixed connective tissue disorder (3 patients) and myositis (1 patient)

**Percentage based on the number of followed up patients at risk (patients with pneumo-renal syndrome at admission and with follow-up available)

*Abbreviations: ANCA* anti-neutrophil cytoplasmic antibody, *ARDS* acute respiratory distress syndrome, *DAH* diffuse alveolar hemorrhage, *GC* gluco-corticoid, *ICU* intensive care unit, *LDH* lactate dehydrogenase, *NA* not applicable, *PLEX* plasma exchange, *SAPS* Simplified Acute Physiology Score

### Comparison between patients with connective tissue disorder and vasculitis

A diagnosis of autoimmune disease was made 5 [2–11] days after hospital admission and 0 [− 1 to 2] days after ICU admission. A small-vessel vasculitis was diagnosed in 79 (76%) patients, and a connective tissue disorder in 25 (24%). Organ involvement of the autoimmune disease was histologically confirmed in 49 (47%) cases. Comparison between patients with connective tissue disorder and vasculitis is shown in Table 1. Patients with vasculitis were older (61 [39–71] vs. 42 [27–55] years old, *p* = 0.003) and were more often males (61% vs. 29%, *p* = 0.01). Pneumo-renal syndrome was more frequent in patients with a vasculitis diagnosis (89% vs. 56%, *p* = 0.0009). Dyspnea evolution was more acute in patients with a connective tissue disorder (time from dyspnea onset to ICU admission 1 [0–10] day vs. 6 [1–24] days, *p* = 0.018). Patients with a connective tissue disorder had lower platelet counts and higher LDH levels on ICU admission.

All patients but one received a specific treatment based on steroids, and 93 (89%) patients received adjunctive steroid pulse therapy. An immunosuppressive therapy based on cyclophosphamide or rituximab was initiated in 72 (69%) and 12 (11.5%) patients, respectively. Cyclophosphamide and rituximab were associated in 6 (6%) patients. PLEX was initiated in 57 (55%) patients. Patients who received PLEX tended to be more severe at ICU admission (SAPS II: 37.5 [28–49.5] vs. 32.5 [22–41.75], *p* = 0.073). In univariate analysis, there was no difference in mortality between patients treated or not with PLEX (supplementary eTable-2).

### Invasive mechanical ventilation requirement, weaning, and ICU management

**Fig. 1:**
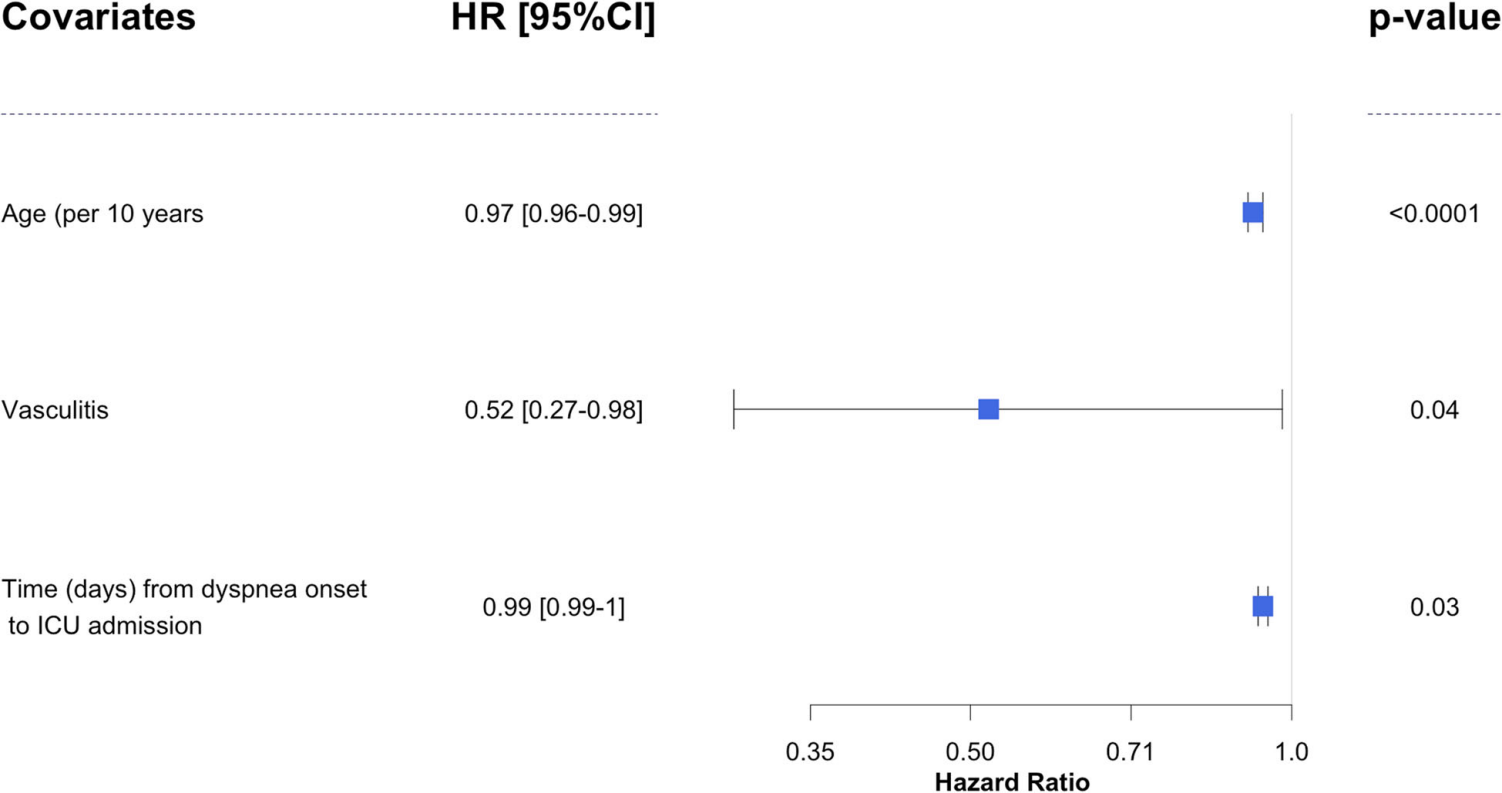
Multivariate analysis of factors associated with mechanical ventilation weaning

Noninvasive mechanical ventilation was implemented in 33 (32%) patients, failing in 18 (55%) who were subsequently intubated. Invasive mechanical ventilation was required in 52 (50%) patients overall, of whom all fulfilled the ARDS criteria. Patients were intubated 0 [0–2] days after ICU admission. Univariate analysis of factors associated with mechanical ventilation requirement is displayed in Table 2. Duration of mechanical ventilation was 12 [6–22] days. Univariate analysis of factors associated with mechanical ventilation weaning is shown in supplementary e-Table 3. In multivariate analysis, factors associated with a longer mechanical ventilation weaning were age (HR [95%CI] 0.97 [0.96–0.99] per 10 years, *p* < 0.0001), vasculitis (0.52 [0.27–0.98], 0.04), and time from dyspnea onset to ICU admission (0.99 [0.99–1] per day, *p* = 0.03) (Fig. 1). The median ICU length of stay was 11 [7–18.8] days.

**Table 2:** Univariate analysis of factors associated with mechanical ventilation requirement

Parameters	No invasive ventilation ***n*** = 52	Invasive ventilation ***n*** = 52	***p*** value
Age, years, median [IQR]	50 [29–62]	58 [39–70]	0.12
Charlson comorbidity index, median [IQR]	3 [1.3–4.8]	3 [1–4]	0.59
Vasculitis, yes, *n* (%)	38 (73%)	41 (79%)	0.65
Hemoptysis, yes, *n* (%)	30 (58%)	23 (44%)	0.24
Acute respiratory failure, *n* (%)	28 (54%)	50 (96%)	< 0.0001
Renal involvement, yes, *n* (%)	40 (77%)	43 (84%)	0.46
SAPS II, median [IQR]	29 [19–37]	44 [33–56]	< 0.0001
PaO2/FiO2 ratio, median [IQR]	205 [123–263]	108 [75–190]	0.0003
Time (days) from dyspnea to ICU admission, days, median [IQR]	6 [1–23]	5 [1–16]	0.70
Hemoglobin at day 1, median [IQR]	8.3 [7.4–9.5]	8.6 [7.2–9.5]	0.85
Neutrophil count at day 1, median [IQR]	8.2 [6.4–10.2]	10.6 [7.6–18.0]	0.05
LDH at day 1, UI/L, median[IQR]	432 [317–626]	679 [409–1213]	0.010

*Abbreviations: CI* confidence interval, *HR* hazard ratio, *ICU* intensive care unit, *SHR* subdistribution hazard ratio

### Factors associated with ICU survival

**Fig. 2:**
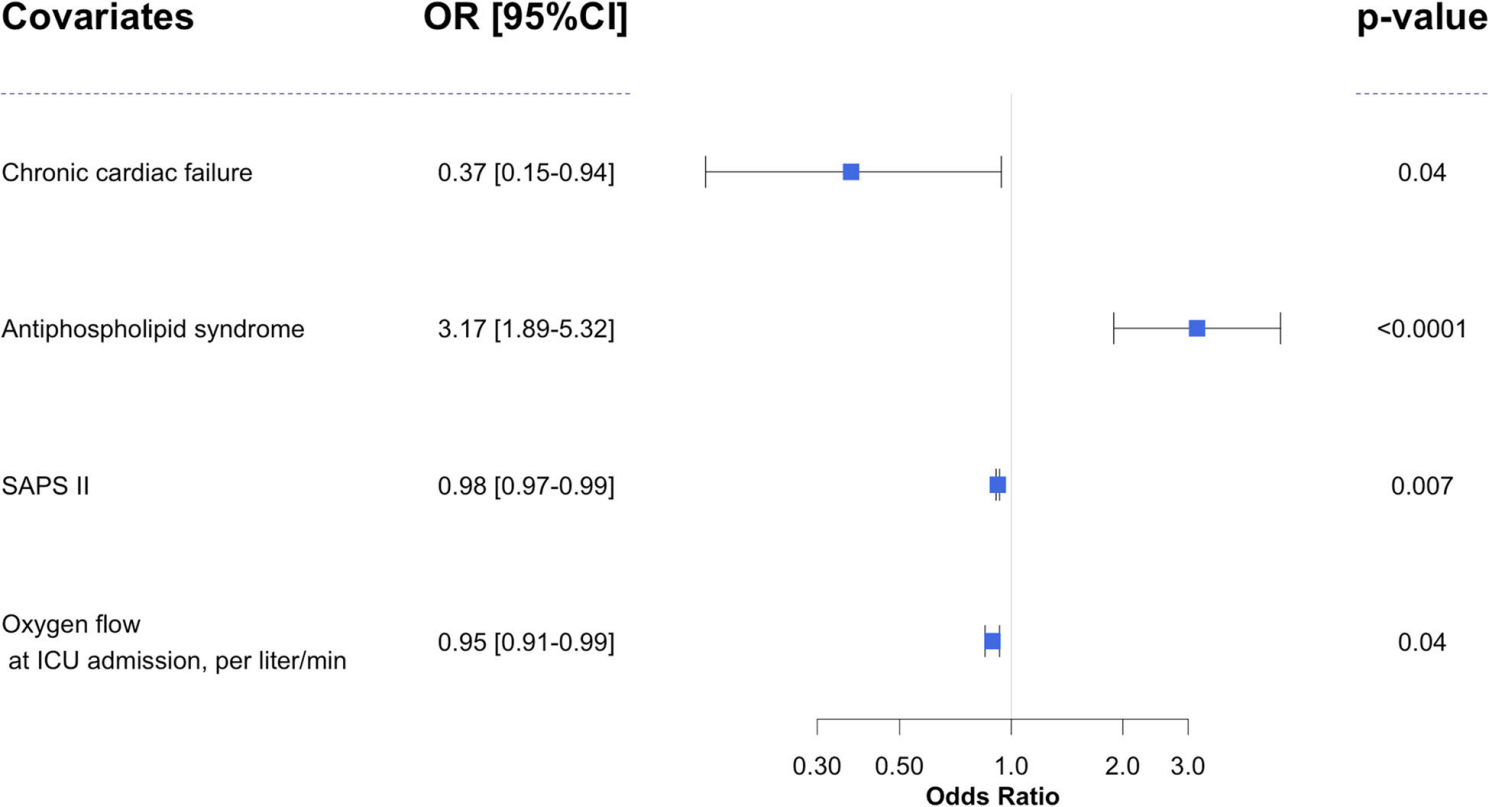
Multivariate analysis of factors associated with ICU survival

Sixteen (15%) patients died in ICU. Death was attributed directly to refractory autoimmune disease in 5 (31%) cases, to refractory ARDS in 4 (25%) cases, to multi-organ failure in 4 (25%) cases, and to septic shock in 3 (19%) cases. In univariate analysis, factors associated with ICU mortality are displayed in Table 3 and supplementary e-Table 4. In multivariate analysis, factors associated with alive status at ICU discharge were chronic heart failure (HR [95%CI] 0.37 [0.15–0.94], *p* = 0.037), APS-related DAH (3.17 [1.89–5.32], *p* < 0.0001), SAPS II (0.98 [0.97–0.99], *p* = 0.007), and oxygen flow at ICU admission (0.95 [0.91–0.998], *p* = 0.04) (Fig. 2).

**Table 3:** Univariate analysis of ICU survival associated factors

Parameter	Univariate analysis	
	SHR 95%CI	***p*** value
Age (per 10 years)	0.87 (0.79–0.95)	0.0016
Sex	1.12 (0.75–1.7)	0.58
Charlson comorbidity index	0.88 (0.81–0.97)	0.0078
Chronic respiratory failure	0.73 (0.29–1.82)	0.49
Chronic cardiac failure	0.34 (0.16–0.74)	0.0063
Chronic renal failure	0.59 (0.3–1.17)	0.13
Steroid treatment	0.78 (0.44–1.37)	0.38
Connective tissue disorder	1.35 (0.82–2.22)	0.24
Systemic lupus	1.07 (0.48–2.41)	0.86
Antiphospholipid syndrome	2.26 (1.35–3.78)	0.0018
Other systemic disease	0.87 (0.36–2.09)	0.76
Vasculitis	1.35 (0.82–2.22)	0.24
ANCA-associated vasculitis	0.95 (0.62–1.45)	0.8
Goodpasture syndrome	1.08 (0.7–1.66)	0.73
Cryoglobulin-associated vasculitis	0.17 (0.02–1.68)	0.13
IgA-associated vasculitis	0.49 (0.15–1.6)	0.24
Relapse	1.07 (0.63–1.81)	0.81
Hemoptysis	1.18 (0.79–1.77)	0.42
Acute respiratory failure	0.57 (0.37–0.89)	0.014
Renal involvement	0.73 (0.46–1.14)	0.17
Digestive involvement	1.31 (0.45–3.85)	0.62
Nervous system involvement	1.21 (0.78–1.87)	0.39
Joint involvement	0.8 (0.48–1.34)	0.41
Skin involvement	0.81 (0.48–1.36)	0.43
Other systemic disease manifestations	1.1 (0.93–1.3)	0.25
SAPS II	0.97 (0.96–0.99)	< 0.0001
Oxygen flow	0.95 (0.91–0.98)	0.0032
PAO2 over FiO2 ratio (per 10 points)	1.02 (1–1.04)	0.0099
Time (days) from dyspnea onset to ICU admission	(0.98–1)	0.13
Time (days) from hospital admission to ICU admission	1.01 (0.98–1.04)	0.6
Hemoglobin at ICU admission	0.97 (0.89–1.05)	0.41
Lymphocytes at ICU admission	2.51 (1.6–3.95)	< 0.0001
Neutrophil count at ICU admission	1.02 (0.98–1.06)	0.45
Platelets count at ICU admission (per 10G/L)	1.01 (0.99–1.02)	0.36
Creatinine at ICU admission (per 10 μmol/L)	1 (0.99–1.01)	0.72
Urine protein/creatinine ratio	0.97 (0.46–2.02)	0.93
LDH at ICU admission (per 100UI/L)	0.98 (0.94–1.02)	0.31
Vasopressor use	0.29 (0.18–0.47)	< 0.0001

*Abbreviations: CI* confidence interval, *HR* hazard ratio, *ICU* intensive care unit, *SAPS* Simplified Acute Physiology Score, *SHR* cause specific hazard ratio

After ICU discharge, 48 (55%) patients were followed up with a median duration of 19 [8–38] months. At the end of follow-up, 22 (65%) of these patients had a chronic kidney disease and 8 (24%) still required renal replacement therapy. Twenty-two (47%) patients had a lung CT-scan evaluation during follow-up showing lung fibrosis for 8 (36%) patients. Pulmonary function testing was performed in 15 (32%) patients and showed a restrictive pulmonary disorder in 6 (40%) cases.

## Discussion

In this multicenter nationwide French study, we report the largest study assessing the outcome and prognosis factors associated with severe DAH related to autoimmune diseases. The conclusions drawn by this study are that (1) ICU mortality was 16% reaching 31% in patients requiring mechanical ventilation; (2) mechanical ventilation was required in half of the cases, of whom all met ARDS criteria; and (3) systemic vasculitis was associated with longer mechanical ventilation duration.

Other studies have reported management and outcomes of patients with DAH, but they have described patients with less severe diseases. Cartin-Ceba et al. have reported outcomes of patients with AAV-related DAH [10]. Some of these patients were asymptomatic and were not hospitalized. Other patients in this study required ICU admission, but they were less severe than in our cohort. Another cohort included patients with variable severity, and DAH was not confirmed by bronchoalveolar lavage in all cases [25].

In the present study, overall ICU mortality was 16% and reached 31% of patients who required mechanical ventilation. Mortality from DAH has reduced during the past years, and the mortality in our cohort is in line with recent DAH cohorts [26, 27]. Major advances in the care of critically ill patients could explain these results. Our results point out that severity at ICU admission is directly linked to ICU mortality. Chronic heart failure has already been reported as associated with a poorer prognosis in patients with DAH [3]. DAH related to autoimmune disease is considered to have a better prognosis compared to non-immune causes of DAH [3, 28]. This may illustrate the benefit of early aggressive immunosuppressive therapies in this context. However, there is no data comparing prognosis of DAH according to autoimmune diseases. In our study, APS diagnosis was associated with a better outcome. Previous studies of APS-related DAH reported good outcomes when DAH was controlled [9, 29]. Although our study was not designed to address this question, neither immunosuppressants nor PLEX therapy seemed to improve prognosis.

Half of the patients required invasive mechanical ventilation. Comorbidities and type of autoimmune disease were not risk factors for mechanical ventilation. These results are in agreement with previous studies, suggesting that the underlying medical context is no longer significantly associated with the risk for intubation after adjustment on the severity of the acute disease [30–33]. Age and small-vessel vasculitis were associated with a longer duration of mechanical ventilation. Patients with small-vessel vasculitis required more frequently renal replacement therapy which could explain in part a negative impact on ventilation weaning [34]. Early ICU admission after dyspnea onset was associated with a shorter duration under mechanical ventilation. Dumas et al. already reported that direct ICU admission was associated with better outcomes in a large study of critically ill patients with systemic rheumatic disease [5].

Our study has several limitations. First, given the retrospective design over a long period of time, supportive care practices may have changed throughout the study period and influenced the results. Second, for the diagnosis of DAH, we used a database encoded by physicians at patient ICU discharge and we cannot exclude that some patients with autoimmune disease-related DAH had been missed. Third, we mixed patients with different autoimmune diseases which mean different prognosis and different management. However, most patients were admitted before autoimmune disease diagnosis. This study might provide some practical data to initially manage these patients with a DAH and a suspected autoimmune disease.

## Conclusion

In conclusion, DAH related to autoimmune disease is a rare but potentially dreadful complication that requires mechanical ventilation in half of the cases admitted to ICU. Age, small-vessel vasculitis, and the time from dyspnea onset to ICU admission were associated with a longer mechanical ventilation duration. The best initial regimen remains to be determined.

## Supplementary information

**Additional file 1: Table S1.** supplementary data on patients with DAH and according to the presence of a vasculitis or a connective tissue disorder. **Table S2.** univariate analysis according to plasma exchange therapy. **Table S3.** Univariate analysis of factors associated with mechanical ventilation weaning. **Table S4.** Univariate analysis of respiratory and vital status outcomes according to diffuse alveolar hemorrhage treatment.

## Data Availability

Not available.

## Abbreviations

ARDS: Acute respiratory distress syndrome
ANCA: Anti-neutrophil cytoplasmic antibodies
APS: Antiphospholipid syndrome
DAH: Diffuse alveolar hemorrhage
ICU: Intensive care unit
LDH: Lactate dehydrogenase
PLEX: Plasma exchange therapy
SAPS II: Simplified Acute Physiology Score
SLE: Systemic lupus erythematosus
