# Supplementary material for: Severe diffuse alveolar hemorrhage related to autoimmune disease: a multicenter study
Source: Crit Care. 2020 May 18;24:231. doi: 10.1186/s13054-020-02936-0 (PMC7236262; doi:10.1186/s13054-020-02936-0)
Supplement: Supplementary file 1 — Additional file 1: Table S1. supplementary data on patients with DAH and according to the presence of a vasculitis or a connective tissue disorder. Table S2. univariate analysis according to plasma exchange therapy. Table S3. Univariate analysis of factors associated with mechanical ventilation weaning. Table S4. Univariate analysis of respiratory and vital status outcomes according to diffuse alveolar hemorrhage treatment. [file 13054_2020_2936_MOESM1_ESM.docx]

**Supplementary e-Table1: supplementary data on patients with DAH and according to the presence of a vasculitis or a connective tissue disorder.**

|  | **All patients (n=104)** | **Connective tissue disorder (n=25)** | **Vasculitis (n=79)** | **p** |
| --- | --- | --- | --- | --- |
| **Comorbidities**, n (%) |  |  |  |  |
| Tobacco smokers | 3 [1-4] | 3 (13%) | 40 (51%) | 0.0008 |
| Hypertension | 31 (30%) | 4 (16%) | 27 (34%) | 0.13 |
| Chronic respiratory failure | 7 (7%) | 2 (8%) | 5 (6%) | 0.66 |
| Chronic cardiac failure | 10 (10%) | 1 (4%) | 9 (11%) | 0.45 |
| Chronic renal failure | 14 (14%) | 3 (13%) | 11 (14%) | 1 |
| Diabetes mellitus | 12 (10%) | 2 (8%) | 10 (13%) | 0.73 |
| Previous steroid treatment | 26 (25%) | 10 (40%) | 16 (20%) | 0.064 |

**Supplementary e-Table 2: univariate analysis according to plasma exchange therapy.**

| **Variables** | **No plasma exchange**  **N=47** | **Plasma exchange**  **N=57** | **p-value** |
| --- | --- | --- | --- |
| Age | 50 [34-69] | 60 [28-67] | 0.92 |
| Charlson | 2.5 [1-4] | 3 [1-4] | 0.83 |
| Connective tissue disorders | 18 (38%) | 6 (11%) | 0.001 |
| Vasculitis | 28 (60%) | 51 (90%) | 0.0005 |
| Systemic disease manifestations |  |  |  |
| Hemoptysis | 23 (49%) | 30 (53%) | 0.84 |
| Renal | 33 (70%) | 50 (89%) | 0.023 |
| ICU admission |  |  |  |
| Acute respiratory failure | 37 (79%) | 74 (72%) | 0.5 |
| SAPS II | 33 [22-42] | 38 [28-50] | 0.073 |
| PaO2/FiO2 ratio | 161 [106-230] | 134 [75-222] | 0.32 |
| Hemoglobin, g/dL | 8.8 [7.5-9.5] | 8.3 [7.2-9.4] | 0.34 |
| ICU management |  |  |  |
| Vasopressor use | 15 (32%) | 21 (37%) | 0.68 |
| Renal replacement therapy | 15 (32%) | 40 (70%) | 0.0002 |
| Mechanical ventilation | 22 (47%) | 30 (53%) | 0.69 |
| Hospital mortality | 6 (13%) | 10 (18%) | 0.59 |

*Abbreviations: ICU: Intensive Care Unit, SAPS II: Simplified Acute Prognosis Score*

**Supplementary e-Table3: Univariate analysis of factors associated with mechanical ventilation weaning:**

| **Variables** | **Univariate analysis** | |
| --- | --- | --- |
|  | **SHR IC95** | **p-value** |
| Age (per 10 years) | 0.98 (0.97-0.99) | 0.0037 |
| Charlson comorbidity index | 0.88 (0.77-1) | 0.042 |
| Chronic cardiac failure | 0.39 (0.17-0.9) | 0.028 |
| Chronic respiratory failure | 0.76 (0.22-2.63) | 0.66 |
| Chronic renal failure | 0.49 (0.21-1.13) | 0.093 |
| Connective tissue disorder | 2.09 (0.96-4.56) | 0.063 |
| Hemoptysis | 1 (0.55-1.83) | 0.99 |
| Acute respiratory failure | (0.21-4.89) | 1 |
| Renal involvement | 0.52 (0.25-1.06) | 0.071 |
| Digestive involvement | 2.05 (0.63-6.69) | 0.23 |
| Nervous system involvement | 2.26 (0.94-5.45) | 0.07 |
| Joint involvement | 0.54 (0.26-1.13) | 0.1 |
| Skin involvement | 0.74 (0.37-1.45) | 0.38 |
| SAPS II | 0.98 (0.97-1) | 0.084 |
| PAO2 over FiO2 ratio (per 10 points) | 1 (1-1) | 0.63 |
| Time (days) from dyspnea onset to ICU admission | 1 (1-1) | 0.0079 |
| Time (days) from hospital admission to ICU admission | 1.01 (1-1.01) | 0.043 |
| Hemoglobin at ICU admission | 0.97 (0.85-1.11) | 0.64 |
| Lymphocytes at ICU admission | 3.12 (1.47-6.63) | 0.0032 |
| Neutrophil count at ICU admission | 1.04 (1.01-1.07) | 0.011 |
| Platelets count at ICU admission | 1 (1-1) | 0.61 |
| Creatinine at ICU admission (per 10µmol/L) | 1 (1-1) | 0.93 |
| Urine protein/creatinine ratio | 1.72 (0.15-19.92) | 0.66 |
| LDH at ICU admission (per 100UI/L) | 1 (1-1) | 0.23 |
| Vasopressor requirement at day 1 | 0.69 (0.41-1.16) | 0.16 |

*Abbreviations: ICU: Intensive Care Unit, LDH: Lactate DesHydrogenase, PLEX: Plasma Exchange, SAPS II: Simplified Acute Prognosis Score*

**Supplementary e-Table 4: Univariate analysis of respiratory and vital status outcomes according to diffuse alveolar hemorrhage treatment.**

| **Treatment** | **No invasive ventilation**  **n=52** | **Invasive ventilation**  **n=52** | **p-value** | **Alive at ICU discharge**  **n=88** | **Dead at ICU discharge**  **n=16** | **p-value** |
| --- | --- | --- | --- | --- | --- | --- |
| Steroids, n (%) | 51 (98%) | 52 (100%) | 1 | 87 (99%) | 16 (100%) | 1 |
| Steroids pulse therapy, n (%) | 46 (89%) | 47 (90%) | 1 | 83 (94%) | 10 (63%) | 0.002 |
| Cyclophosphamide, n (%) | 37 (71%) | 35 (67%) | 0.83 | 64 (73%) | 8 (50%) | 0.083 |
| Rituximab, n (%) | 3 (6%) | 9 (17%) | 0.12 | 7 (8%) | 5 (31%) | 0.019 |
| Plasma exchange therapy, n (%) | 27 (52%) | 30 (58%) | 0.69 | 47 (53%) | 10 (63%) | 0.59 |
| Number of plasma exchange sessions, median [IQR] | 5 [3-7] | 7 [7-12] | 0.001 | 7 [5-9] | 6 [3-10] | 0.57 |

*Abbreviations: ICU: Intensive Care Unit*
